# Erenumab and Possible CGRP Effect on Chronotype in Chronic Migraine: A Real-Life Study of 12 Months Treatment

**DOI:** 10.3390/jcm12103585

**Published:** 2023-05-21

**Authors:** Laura Pilati, Angelo Torrente, Salvatore Di Marco, Salvatore Ferlisi, Giulia Notaro, Marika Romano, Paolo Alonge, Lavinia Vassallo, Ludovica Ferraù, Massimo Autunno, Rosario Grugno, Cecilia Camarda, Filippo Brighina

**Affiliations:** 1 Department of Biomedicine, Neurosciences and Advanced Diagnostics (Bi.N.D.), University of Palermo, 90127 Palermo, Italy; laura.pilati.91@gmail.com (L.P.); dimarcosal@gmail.com (S.D.M.); salvatore9.ferlisi1@gmail.com (S.F.); notarogiuli@gmail.com (G.N.); marika.romano2@gmail.com (M.R.); alongep95@gmail.com (P.A.); lavinia.v1994@gmail.com (L.V.); filippobrighina@gmail.com (F.B.); 2 Headache Center “Casa della Salute Cittadella San Rocco”, Azienda Unità Sanitaria Locale di Ferrara, 44121 Ferrara, Italy; 3 Azienda Sanitaria Locale di Collegno e Pinerolo, 10093 Collegno, Italy; 4 U.O.S.D. Stroke Unit, A.O.U. “G. Martino”, 98124 Messina, Italy; ludo.ferrau@hotmail.it; 5 Centro Regionale Cefalee, U.O.C. Neurologia e Malattie Neuromuscolari, A.O.U. “G. Martino”, 98124 Messina, Italy; mautunno@unime.it; 6 U.O.C. Neurologia, I.R.C.C.S. Centro Neurolesi “Bonino Pulejo”, 98124 Messina, Italy; rosario.grugno@irccsme.it

**Keywords:** migraine, CGRP, erenumab, sleep, circadian rhythm, real life

## Abstract

The introduction of monoclonal antibodies (mAbs) directed against the calcitonin gene-related peptide (CGRP), or its receptor (CGRPr), revolutionized migraine management due to their high efficacy and few side effects. Data suggest that the CGRP may even be implicated in circadian rhythm, but studies about the effect of anti-CGRP treatments on sleep are still lacking. The aim of the present study was to assess the effect of erenumab (70 and 140 mg per month), a human mAb directed against CGRPr, on chronotype in chronic migraineurs; secondly, we assessed its efficacy, safety, and the effects on anxiety and depression. Sleep was evaluated using self-administrable questionnaires investigating chronotype, sleep quality, and daytime sleepiness. Migraine diaries and several self-administrable questionnaires regarding headache impact and psychological correlates were evaluated every 3 months during 12 months of treatment. Eighty-eight patients were included; most of them showed a significant reduction in headache frequency and an improvement in psychological symptoms. Moreover, an initial change in chronotype was observed at the three-month assessment from a morning chronotype to an intermediate one; a similar trend remained in the other evaluations, even if it did not reach a statistical significance. Lastly, patients who responded to the treatment showed a progressive sleep efficiency reduction. The present real-life study hypothesized the influence of erenumab on chronotype, representing a link between circadian rhythm, CGRP, and migraine.

## 1. Introduction

Migraine is the primary cause of disability for those under the age of 50 years and is the third most common and second most disabling disease in the world [1], affecting both patients and their families [2]. Despite its significant impact, the available preventive treatments are non-specific [3] and poorly tolerable [4]. In this context, the advent of mAbs against the CGRP or CGRPr represents a new era in migraine management, both for pain and aura symptoms [5,6]. Evidence from randomized controlled clinical trials strongly supports the efficacy and safety of these agents in the prevention of both episodic and chronic migraine (EM, CM) [7,8]. Among anti-CGRP(r) mAbs, erenumab (in monthly doses of 70 or 140 mg), a fully human mAb directed against CGRPr, was the first approved specific preventive treatment for migraine, whose efficacy and safety have been demonstrated in both EM and CM [9,10]. In the context of real-life studies of anti-CGRP(r) mAbs [11,12,13], few have investigated erenumab’s efficacy with a multidisciplinary approach including sleep for 12 months [14,15]. Particularly, an improvement in both subjective and objective sleep quality was demonstrated in patients treated with erenumab [16]. Currently, there are no data about erenumab therapy in CM investigating even circadian rhythm.

Migraine and sleep show a complex and bidirectional relationship; on the one hand, migraineurs often report insomnia due to headache attacks, and, on the other hand, poor sleep quality is one of the main triggers for a migraine attack. Particularly in migraineurs, sleep appears to be characterized by a reduction in quality and efficiency [17]. Moreover, migraine patients often complain of excessive daytime sleepiness and unrefreshing sleep [17]. In addition, sleep deprivation may worsen migraine because it causes an increase in adenosine levels (i.e., a precipitating factor of migraine attacks) or leads to an increased cortical spreading depression susceptibility due to a rise in cortex glutamate levels [18]. Migraine and sleep may find a link even in the neurotransmitters involved in both, such as dopamine, serotonin, norepinephrine, and orexin, which regulate sleep but have an important role also in pain transmission, processing, and modulation [18]. Another sleep-related aspect to consider is the chronotype (i.e., the individual behavioral preference about the time to go to sleep); the morning chronotype appears to be more correlated with CM, so much so that it is defined as a predictor of disease severity [19]. However, several studies did not find a fixed chronotype in migraine sufferers but a greater predisposition to the extreme traits that are the evening or the morning ones [20]. To date, the only experimental data about CGRP and sleep have been derived from Drosophila models, in which animals with a homolog of the CGRP knock-out gene were created. The authors observed that the animals acquired better sleep, especially in the second half of the night [21]. Studies on humans are lacking, but there is evidence that anti-CGRP therapy can influence sleep [22].

The present multicenter study investigated the effect of erenumab on subjective sleep quality and circadian rhythm, hypothesizing that the CGRP represents the link between them. The primary endpoint of our study was to assess the influence of erenumab on the sleep quality and on the chronotype in migraineurs. Secondly, the efficacy of the treatment was evaluated through the reduction of monthly migraine days (MMDs) in a period of 12 months. Among the secondary endpoints, there was also a comparison of the different dosages used in terms of efficacy, safety, and reduction in monthly acute medication intake. Finally, the effect of erenumab on anxiety and depression was evaluated.

## 2. Materials and Methods

### 2.1. Population

Patients affected by CM evaluated at the Headache Centers of “Paolo Giaccone” General University Hospital in Palermo, I.R.C.S.S. Centro Neurolesi “Bonino Pulejo”, and “Gaetano Martino” General Hospital in Messina, Italy, were included in the study. All patients were recruited from January 2021 to February 2022.

The diagnosis of CM was made according to the criteria of the International Classification of Headache Disorders, 3rd edition (ICHD-3) [23], as was the diagnosis of medication overuse [24]. At the time of the study, there were no established criteria for reimbursement, and the administration of erenumab was guaranteed by the supply of the producing company. According to the eligibility criteria for the treatment established by the European Headache Federation [25] and the American Headache Society [26], patients who had failed at least 3 migraine preventive treatments were included. Patients with any history of cardiovascular or psychiatric diseases were excluded, as in recent clinical trials. Moreover, we excluded patients with other primary or secondary headaches or those affected by other neurological or chronic painful conditions, such as fibromyalgia. Lastly, patients with any reported active sleep disorder were also excluded.

Patients underwent erenumab treatment for up to 12 months. Withdrawal was possible in the case of severe adverse events, lack of compliance, ineffectiveness (i.e., reduction < 30% from baseline MMDs), or patients’ decision. At baseline, other concomitant pharmacological preventatives were continued without any dose modification, and, in the case of drug overuse, we did not suggest any detoxification, according to current recommendations [25]. During the study period, it was then possible to discontinue previous concomitant migraine preventatives in the case of good efficacy from erenumab. 

### 2.2. Study Design and Data Collection

We performed a 12-month, real-life study of erenumab on CM. The following data were collected: demographic and clinical patients’ characteristics such as current migraine treatment, comorbidities, and MMDs. Patients were asked to keep note of every migraine attack using diaries. Particularly, during the study period, we investigated changes in MMDs (i.e., the number of days in which a patient showed a headache with migraine characteristics or took an acute drug for headache with its resolution), distinguishing patients as non-responders (i.e., reduction from baseline MMDs < 30%) or responders (i.e., MMDs reduction ≥ 30%). Among responders, we distinguished 30%, 50%, 75%, and 100% responders based on the reduction in MMDs compared to baseline (i.e., 30% responders were those ones who had a reduction in MMDs of between 30 and 49% and so on). Moreover, we investigated the use of symptomatic drugs (i.e., analgesics or triptans intake), analyzing the number of days with symptomatic medications per month (DSMs). Erenumab was administered subcutaneously every 28 days at a dose of 140 mg (i.e., two 70 mg injections) or 70 mg. There was the possibility to switch from the 70 mg dosage to the 140 mg one in the case of no efficacy (i.e., non-responders) evaluated at week 12.

Regarding sleep, a subjective evaluation of the patients’ chronotype, sleep quality, and sleepiness was performed, using self-administered scales. The investigation of the chronotype was performed using the Morningness–Eveningness Questionnaire—self-assessment version (MEQ-SA), dividing patients into the evening (MEQ between 16 and 41), morning (MEQ > 59), and intermediate (MEQ between 41 and 59) chronotypes [27]. Furthermore, sleep quality was investigated using the Pittsburgh Sleep Quality Index (PSQI) [28] and Sleep Condition Indicator (SCI) [29], while daytime sleepiness was studied using the Epworth Sleepiness Scale (ESS) [30]. Sleep data were investigated from just one of the three centers joining the study.

Moreover, each patient was tested with the following self-administrable questionnaires for migraine impact on life and psychiatric comorbidity: the Migraine Disability Assessment Scale (MIDAS) [31], Headache Impact Test, 6th edition (HIT-6) [32], and Beck Depression Inventory (BDI) [33].

**Figure 1:**
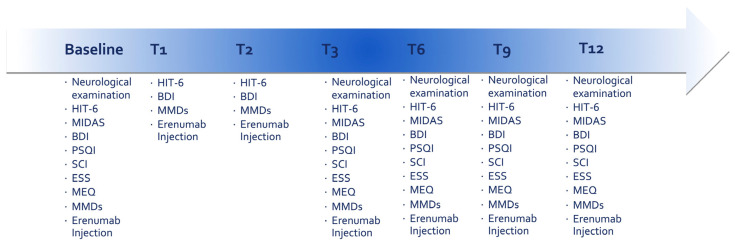
Timeline of study design. Abbreviations: BDI = Beck Depression Inventory; ESS = Epworth Sleepiness Scale; HIT-6 = Headache Impact Test, 6th edition; MIDAS = Migraine Disability Assessment Scale; MMDs = Monthly Migraine Days; MEQ = Morningness–Eveningness Questionnaire; PSQI = Pittsburgh Sleep Quality Index; SCI = Sleep Condition Indicator.

The above-mentioned data were assessed at different times: baseline (T0) and every 3 months after the treatment start (T3, T6, T9, and T12). MMDs, DSMs, HIT-6, and BDI scores were evaluated every month after the treatment start for the first three months (T1, T2, and T3). Furthermore, a neurological examination was performed every 3 months (see Figure 1). Due to the multicenter nature of the study, we expected some of the collected data to be heterogeneous.

### 2.3. Objectives

The primary endpoints constituted the variation in the scores of the MEQ-SA, PSQI, SCI, and ESS questionnaires. Secondly, we investigated the changes in MMDs, DSMs, and responders’ rates. Moreover, changes in MIDAS, HIT-6, and BDI scores were recorded. Finally, safety outcomes included the presence of any adverse event, including serious ones (i.e., leading to hospitalization, death, or treatment discontinuation). Among the secondary endpoints, there was also a comparison of the different dosages used in terms of efficacy, safety, and reduction in DSMs. 

### 2.4. Statistical Analysis

Categorical data are reported as absolute frequencies and percentages, while continuous data are reported as mean ± standard deviation (SD); scores of scales are reported as median and interquartile range (IQR). We used IBM SPSS statistical software and performed the Mann–Whitney U test for independent samples to compare continuous variables. Statistical significance was set at *p* < 0.05.

## 3. Results

### 3.1. Population

Eighty-eight CM patients were included, of whom 30, 57, 70, and 88 completed 12, 9, 6, and 3 months of follow up, respectively, at the time of data analysis. Patient characteristics are shown in Table 1. At baseline, 84 (95%) patients showed criteria for drug overuse. All patients presented multiple previous preventatives failures with an average of 4 ± 1.6 drugs. Particularly, 76 patients (86%) reported failures of antiepileptics, 78 (89%) antidepressants, 51 (58%) onabotulinumtoxinA, 47 (53%) calcium antagonists, 52 (59%) beta-blockers, and 6 (7%) other preventive treatments such as transcranial magnetic stimulation. During the treatment period, according to clinicians’ decisions, eight patients (9%) discontinued the previous oral treatments used because of the brilliant efficacy obtained from erenumab, without showing differences compared to the other responders.

Regarding the dosage, 38 patients (43%) received 140 mg from baseline, while 50 (57%) started erenumab at a dose of 70 mg. During the whole period, 11 patients (22%) received an increase in the monthly dose (70 to 140 mg) since they were non-responders at T3. Patients who started erenumab at the dose of 140 mg presented higher MMDs at baseline than the ones starting at 70 mg dosage (25.71 ± 5.67 vs. 21.62 ± 5.10, respectively, *p* = 0.001).

### 3.2. Primary Outcomes: Migraine, Sleep, and Erenumab

**Figure 2:**
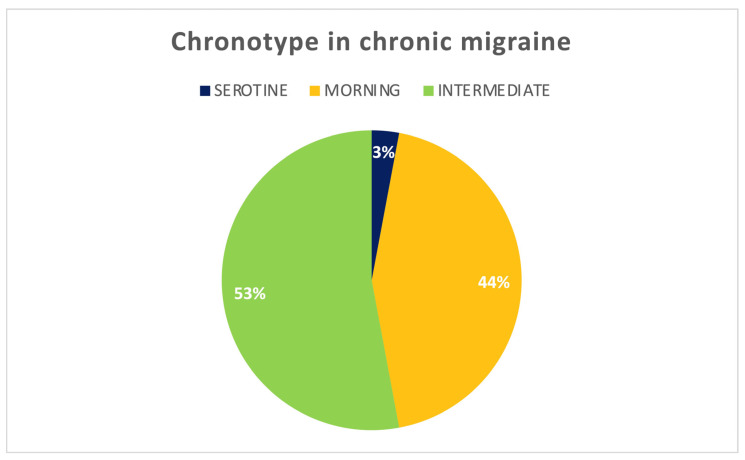
Baseline chronotype of the studied population.

**Figure 3:**
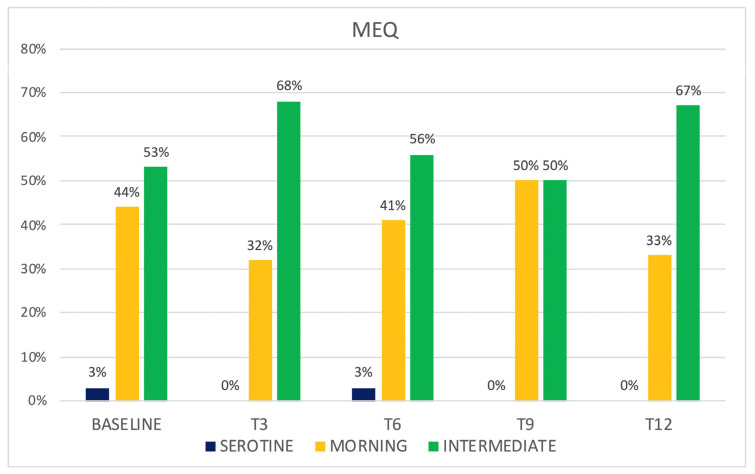
MEQ variation in CM patients treated with erenumab. Abbreviations: CM = Chronic Migraine; MEQ = Morningness–Eveningness Questionnaire.

Patients did not report any known sleep disorders during medical history collection. Sleep was assessed at baseline (Table 2) and during quarterly follow ups in a subpopulation of 34 (39%) patients who underwent the 140 mg dosage. The baseline morning chronotype was shown in 44% of the population, an intermediate one in 53%, and an evening one in 3% of cases (Figure 2). A significant reduction of MEQ was observed between baseline and T3 (*p* < 0.05) from a morning chronotype in favor of an intermediate one (32% vs. 68%) (Figure 3). This variation was not confirmed in the successive follow ups.

**Figure 4:**
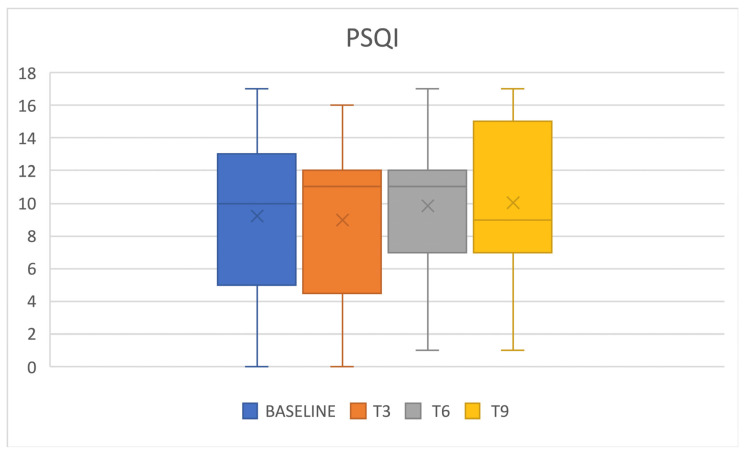
PSQI and erenumab. PSQI = Pittsburgh Sleep Quality Index.

**Figure 5:**
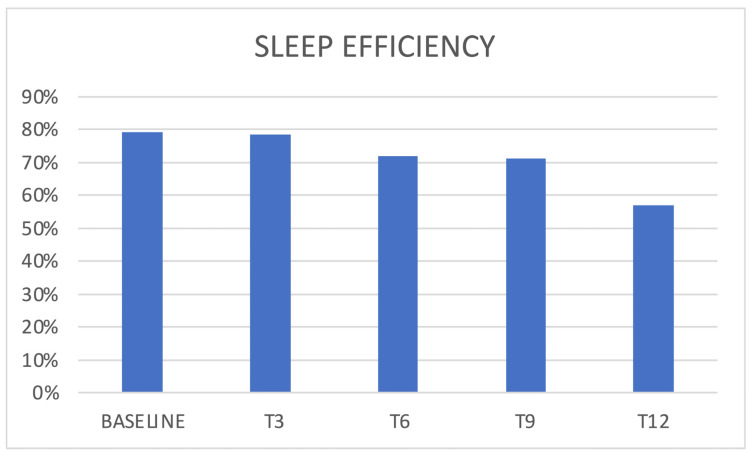
Sleep efficiency and erenumab.

Regarding sleep quality, a PSQI score > 5 (poor sleep quality) was present at baseline in 64% of patients and did not vary significantly at follow up (Figure 4). Sleep efficiency (i.e., the ratio between the hours spent sleeping and the ones spent in bed) showed a slight reduction trend, without reaching statistical significance (Figure 5).

**Figure 6:**
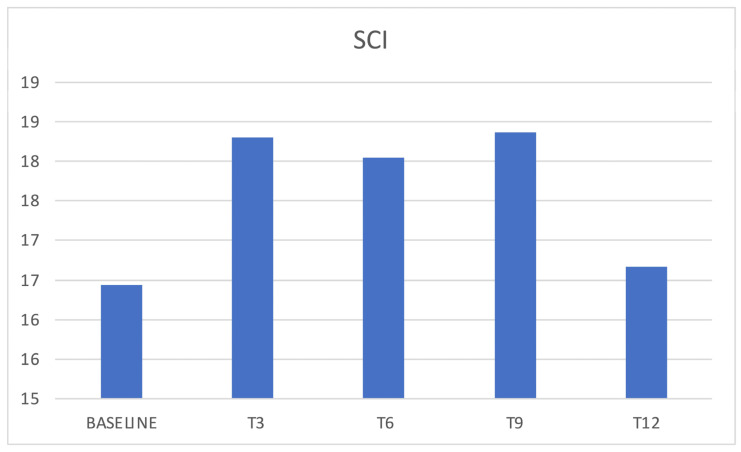
SCI and erenumab. SCI = Sleep Condition Indicator.

SCI score significantly increased at T3 compared to baseline (*p* = 0.0144) even if such an increase was not confirmed during later investigations (*p* > 0.05) (Figure 6). A SCI < 16, which correlates with insomnia, was present at baseline in 62% of patients (Table 2). 

**Figure 7:**
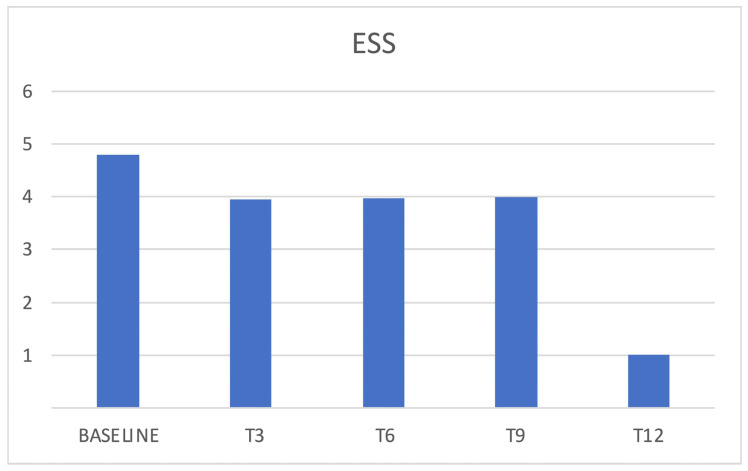
ESS and erenumab. ESS = Epworth Sleepiness Scale.

ESS score showed a slight reduction during follow ups without reaching a statistical significance (Figure 7). An ESS > 10 was present in 15% of patients at baseline.

A post hoc analysis was performed to find any significant change in sleep-related parameters in the subpopulations of responders (n = 29) and non-responders (n = 5). The only parameter that showed a change was the sleep efficiency, which reduced significantly in the responders population: T0 = 78 ± 15%, T3 = 77 ± 10%, T6 = 71 ± 12%, T9 = 70 ± 9%, and T12 = 57 ± 0% (n = 1); the only significant variations were: T6 vs. T0 with *p* = 0.047, T9 vs. T0 with *p* = 0.016, and T9 vs. T3 with *p* = 0.021. No other significant change was observed in the two populations.

### 3.3. Secondary Outcomes

#### 3.3.1. Erenumab Efficacy

**Figure 8:**
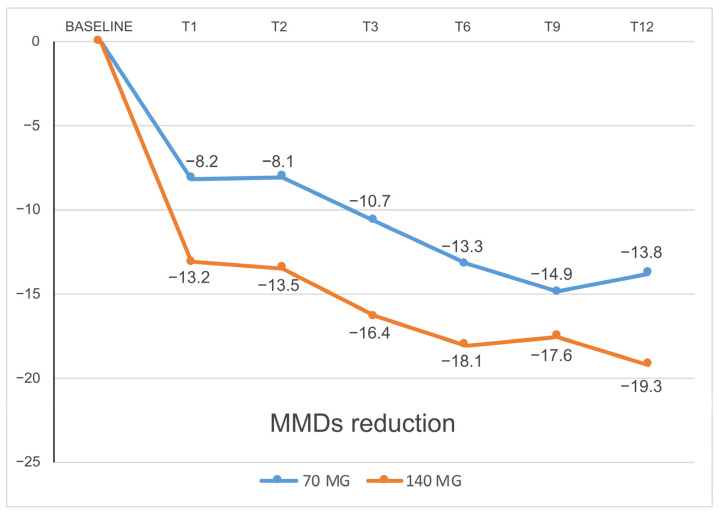
Reduction in MMDs compared to baseline. MMDs = Monthly Migraine Days.

**Figure 9:**
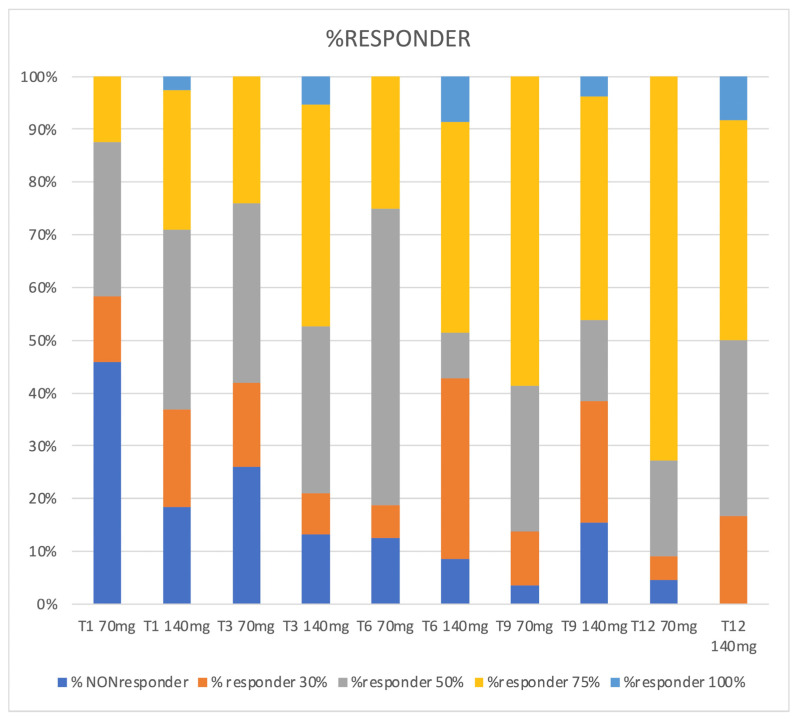
Percentage of responders, 70 mg vs. 140 mg. Note that there is no T12 evaluation responders’ rate for 70 mg dosage since only 140 mg patients reached this follow-up time.

At T3, we recorded a statistically significant reduction in MMDs compared to baseline: from 21.62 ± 5.10 to 10.94 ± 7.87 in patients receiving 70 mg (*p* < 0.001) and from 25.71 ± 5.67 to 9.32 ± 8.75 in those taking 140 mg (*p* < 0.001). Efficacy was maintained in patients who continued treatment after 6, 9, and 12 months. In particular, for patients with the 70 mg dosage, T6 = 8.84 ± 5.95, T9 = 6.72 ± 5.19, and T12 = 8.02 ± 4.24; regarding the different timing comparison, statistical significance was reached for every timing vs. T0 (*p* < 0.001). Moreover, there was a significant difference between T3 and T9 (*p* = 0.014) that was not confirmed in T3 vs. T12 (*p* = 0.766); the other comparisons among the MMDs of T3, T6, T9, and T12 were not statistically significant. As far as the 140 mg dosage was concerned, we obtained: T6 = 7.74 ± 6.87, T9 = 8.21 ± 6.64, and T12 = 10.25 ± 6.70. Even here, efficacy among every timing was confirmed vs. T0 (*p* < 0.001); with such a dosage, no significant difference was observed among T3, T6, T9, and T12. (Figure 8). Efficacy rates, as well as percentage reduction in MMDs, are shown in Figure 9.

**Figure 10:**
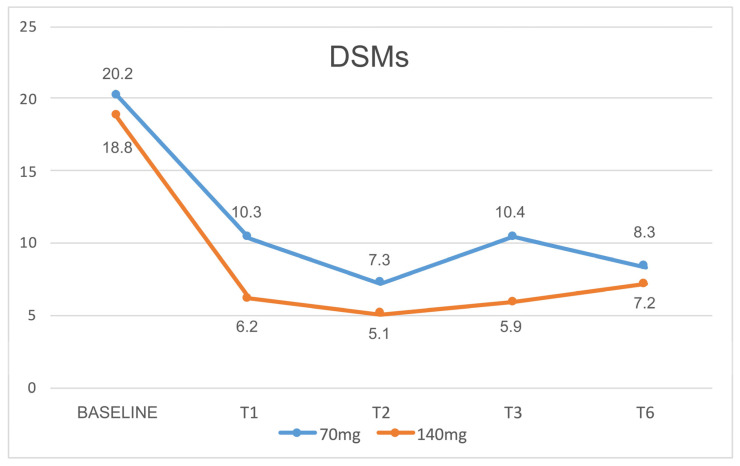
Reduction in DSMs. DSMs = Days with Symptomatic Medications per month.

The mean number of DSMs showed a relevant reduction (*p* < 0.001) from baseline to T3, T6, T9, and T12 months (Figure 10) for both erenumab dosages. So, during follow up, about 80% of patients that showed drug overuse at baseline were detoxified without any specific intervention (DSM decreased to far below the threshold for medication overuse headache).

Moreover, the efficacy of the two dosages and the response time were compared. After the first dose of 70 and 140 mg (T1), 13% and 18% of patients showed a 30% response in terms of MMD reduction, respectively, compared to baseline; 29% and 34% had a 50% response, 13% and 26% had a 75% response, and 0% and 3% had a 100% response, respectively. These percentages increased from month to month, as shown in Figure 8. The dosage of 140 mg initially proved to be more effective than the 70 mg one in reducing MMDs after just the first month (*p* = 0.00410) and up to the sixth month (*p* = 0.00485), then the efficacy became progressively comparable (Figure 2 and Figure 3). No significant differences were found between the two dosages regarding DSMs (*p* > 0.05) (Figure 10).

Eighteen patients (20%) were defined as non-responders at T3, and four of these voluntarily decided to drop out. T3 non-responders showed a higher number of MMDs at baseline (26.1 vs. 22.7; *p* = 0.004) and a higher median number of preventive treatment failures (4.6 vs. 3.9; *p* = 0.042) compared to responders (Table 3). No other baseline characteristic was revealed to be significantly different between the two groups. At T3, we discussed the continuation of treatment directly with non-responders, and 14 of them decided to continue; of them, six patients (43%) became responders at T6, and they maintained the therapeutic goal in the subsequent follow ups (note that four of these patients were among the 11 who started with 70 mg and then received the dose increase at T3). At T9, seven (50%) of the non-responders became responders.

#### 3.3.2. Migraine Impact on Life and Psychological Correlates 

**Figure 11:**
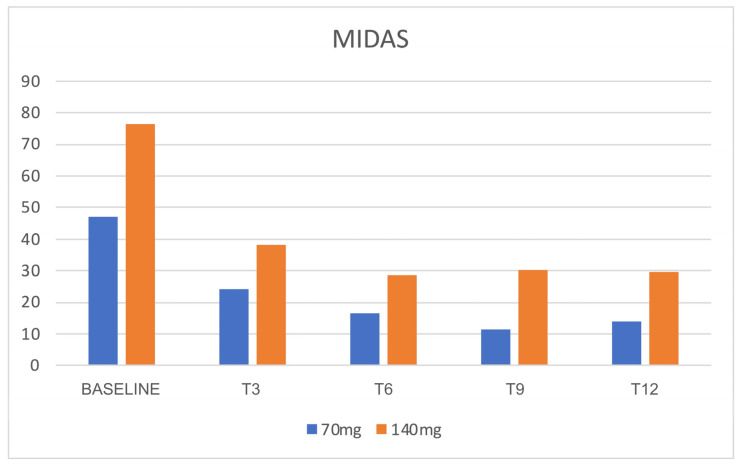
MIDAS scores. N = 88 patients at T0. MIDAS = Migraine Disability Assessment Scale.

**Figure 12:**
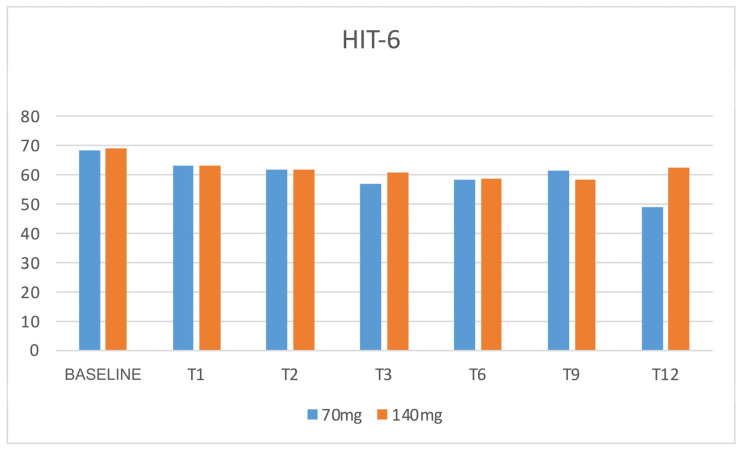
HIT-6 scores. N = 61 patients at T0. HIT-6 = Headache Impact Test, 6th edition.

**Figure 13:**
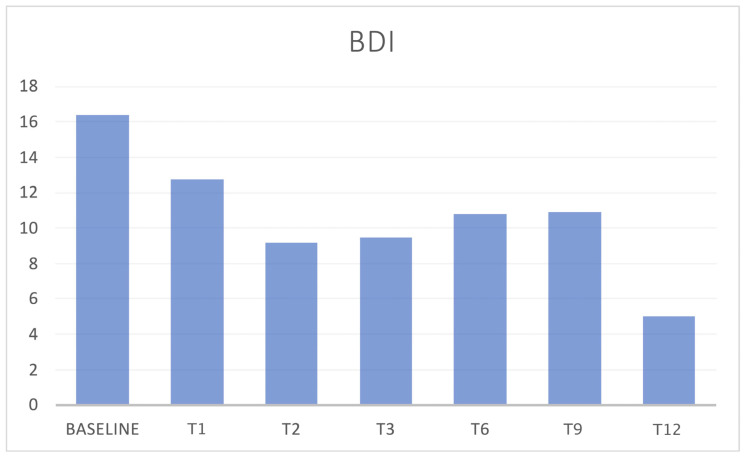
BDI scores. Data are only about the 140 mg population. N = 45 at T0. BDI = Beck Depression Inventory.

During the treatment with both dosages of 70 and 140 mg, patients referred to a minor impact of headache in everyday life, with a concomitant reduction in migraine-associated disability, as shown by the significant reduction in MIDAS and HIT-6 scores during all the evaluations compared to baseline (*p* < 0.05) (Figure 11 and Figure 12). Even the depression-associated symptoms improved, as demonstrated by a reduction in the BDI scores that was reported in a subpopulation of 45 patients treated with 140 mg erenumab (Figure 13).

#### 3.3.3. Adverse Events

We recorded 10 different adverse events in 33 patients (37.5%). The most common adverse event was constipation, which was observed in nine patients (10.2%). No adverse event led to withdrawal. Moreover, five patients (5.7%) complained of insomnia, particularly the night following the administration of the drug. The complete list of adverse events is shown in Table 4.

## 4. Discussion

The present multicenter, real-life study was conducted in a population of chronic resistant migraineurs, a type of patient previously excluded from clinical trials [10,34] but examined subsequently in recent real-world studies [35,36,37].

Migraine and sleep disturbances are common and often burdensome chronic conditions with a high prevalence in the general population. These disorders often coexist, and this has led to the hypothesis of an association not only guided by chance. Indeed, some studies have provided evidence that migraineurs show worse sleep quality than non-migraineurs [38]. On the other hand, poor sleep quality is associated with a higher frequency of migraine attacks. Despite extensive investigations, the exact nature and direction of the association remains enigmatic. Recent biochemical and functional imaging studies have identified central nervous system structures and neurotransmitters involved in the pathophysiology of migraine that are also important for normal sleep regulation. According to the results of a population-based prospective study by Nord-Trøndelag Health [39,40], the association between migraine and insomnia can be bidirectional, and it is not exclusively attributable to anxiety and depression [17]. The pathological mechanism underlying this association is not yet fully understood. According to most of the available studies, the onset of the migraine attack follows a circadian variation, with a morning or night peak of migraine onset [41]. The relationship between the CGRP and sleep or circadian rhythm has been poorly investigated so far, although some effort has been made in other disorders with a more prevalent circadian rhythmicity, such as cluster headache [42]. Currently, only one study, conducted in Drosophila models, showed that “loss of function” of a homolog of the CGRP determined better sleep, especially in the second half of the night [21].

In the presented cohort, baseline data confirmed poor sleep quality in chronic migraineurs, as confirmed by reduced sleep efficiency, in addition to reduced SCI and PSQI scores. We, therefore, evaluated how erenumab acts on sleep; an improvement in sleep quality was expected both by its reduction of the number of headache attacks and by its acting as a “loss of function” of the CGRP. During the treatment with erenumab, there was an initial slight reduction of insomnia with the improvement of SCI scores and a reduction of perceived sleepiness, even if such results were not confirmed in later follow ups, and statistical significance was reached just from SCI score variation. It is possible that the limited number of patients who completed the sleep-related questionnaires (34 out of 88) affected the significance of the results, and perhaps studying a bigger population could result in more relevant findings. Conversely, some patients reported insomnia as an adverse event during the treatment, an event that has not yet been investigated in the literature. Patients reported cognitive hyperarousal, expressed as difficulty in falling asleep during the night following the administration of the drug and within the following week. This dichotomy between the slight improvement in SCI scores and the reported insomnia could be attributable to an acute adverse event that does not affect the quality of monthly sleep. Moreover, patients showed chronotype variation during therapy with erenumab; those who had an MEQ morning chronotype at baseline showed a shift towards the intermediate one. The change in MEQ score did not reach statistical significance in the other follow ups (probably due to the progressive reduction in the number of patients that reached them), but a similar trend may be observed. This result could therefore be attributed to an improvement in headache or to an influence on CGRP pathways affecting the mechanisms underlying the circadian rhythm. From the literature, we know the morning chronotype is more correlated with chronic migraine, so it is considered a predictor of disease [19]. However, several studies did not find a fixed chronotype in migraine sufferers but a greater predisposition to the extreme traits (i.e., the evening or the morning one) [20]. Sleep efficiency was affected as well during erenumab therapy, especially in the responders population, in which a significant reduction was observed after 6 months of therapy. Such a finding may correlate with the insomnia adverse event reported by patients. Nevertheless, general sleep quality was not affected, as PSQI scores did not show any significant variation.

Regarding erenumab efficacy, we recorded higher percentages of patients with a response rate of over 50%, 75%, or even 100% in the reduction of MMDs compared to the available studies [10,34]. This is probably due to the maintenance of the treatment even after an initial non-response at the third month, even though the occurrence of a placebo effect cannot be excluded. Therefore, our data support the continuation of the therapy for 6–9 months before interrupting it if no response is recorded at the third month of treatment. Our data also show that the response to erenumab was persistent in most cases even at one year of treatment. It is important to note that we did not suggest any detoxification for patients who presented acute drug overuse. The response to erenumab, as well as to any migraine treatment, is influenced by the extreme variability of migraine frequency and severity. However, a portion of patients who did not achieve a significant response in terms of MMDs reduction may still have had a significant gain in terms of disability, associated symptoms, and symptomatic drug consumption. We also demonstrated greater efficacy of the dosage of 140 mg compared to 70 mg in the reduction of MMDs compared to the baseline at least in the first 6 months, confirming the greater rapidity of action, as already described in literature [43]. Such data suggest the possibility of using 140 mg in CM cases with greater disability for a faster benefit. A further point of debate is the possibility of increasing the dose from 70 mg to 140 mg per month. In our observational study, the increase of the dose at the third month resulted in greater efficacy. Moreover, since erenumab is an expensive treatment, it is necessary to identify the factors that predict response. In the present study, non-responders presented higher baseline MMDs and a higher number of preventive treatment failures than responders, in agreement with other studies [14]. Predictors of response to anti-CGRP therapy could support the management of CM patients, and studies in the literature suggest that they could be found both in clinical and biological factors [44,45,46].

Additionally, the CM patients included in our study showed a high migraine frequency with a high headache-related impact and disability. Erenumab was able to reduce the impact of headaches in patients’ lives and symptoms of depression and anxiety.

Regarding treatment-related adverse events, we found comparable rates to the available randomized controlled trials. The percentage of patients with constipation was similar in our cohort (10%) compared to that in other Italian studies [14] and higher than that in other countries, probably because patients and their treating physicians expected such an event. However, the constipation was mild and well controlled with diet, and, in all cases, it did not lead to treatment interruption. Among the adverse events that were not present in the technical data sheet, we traced insomnia. In our study, the treatment discontinuation rate was inferior to other ones in literature and mainly due to ineffectiveness [14]. 

The strengths of the present study include the relatively high number of patients compared to other real-life studies and a significantly long follow up, as well as a multidimensional assessment of the of patients treated with erenumab, including assessment of sleep quality and chronotype. Nevertheless, the study presented several limitations: firstly, it was a multicenter study, so some data were heterogeneous (especially regarding the questionnaires used); secondly, the selection of the population was limited to chronic migraineurs, while the inclusion of episodic ones may have revealed more evident changes in chronotype and sleep-related data; thirdly, the sample of patients who completed the sleep-related questionnaires was not large, while wider populations may reveal more consistent results; lastly, an objective evaluation of sleep was not performed, so future studies should consider the use of tools such as actigraphy or salivary melatonin dosage.

## 5. Conclusions

In conclusion, a correlation between erenumab therapy and chronotype was suggested, even if it was not possible to demonstrate whether it depended on headache reduction or on a direct effect of the medication. Insomnia was an adverse event yet it was not described, and the causes should be further investigated. In addition, responders showed a reduction in sleep efficiency without any change in sleep quality. Regarding erenumab efficacy, this real-life study demonstrated how a dose of 140 mg was very effective, especially in the first months of treatment. The effect of erenumab on chronotype has never been studied, and the present work may represent an input for future research regarding the link among circadian rhythm, the CGRP, and migraine. 

## Figures and Tables

**Table 1:** Patients’ baseline characteristics.

Patients (n = 88)	
Women, n (%)	75 (85%)
Age, mean ± SD	50.9 ± 9.80
MMDs, mean ± SD	23.39 ± 5.67
DSMs, mean ± SD	18.26 ± 7.44
Drug overuse patients, n (%)	84 (95%)
Patients with NSAIDs overuse, n (%)	44 (50%)
Triptans overuse patients, n (%)	45 (51%)
No. of failed preventive treatments, mean ± SD	4.05 ± 1.62
Failure to respond to onabotulinumtoxinA, n (%)	51 (58%)
Concomitant use of preventive therapies, n (%)	50 (57%)
Patients who started erenumab 140 mg, n (%)	38 (43%)
Patients who started erenumab 70 mg, n (%)	50 (57%)

Abbreviations: DSMs = Days with Symptomatic Medications per month; MMDs = Monthly Migraine Days; NSAIDs = Non-Steroidal Anti-Inflammatory Drugs.

**Table 2:** Baseline sleep characteristics.

Sleep and Migraine (n = 34)	
MEQ, mean ± SD	56.85 ± 7.60
evening (16–41), n (%)	1 (3%)
morning (> 59), n (%)	15 (44%)
intermediate (42–59), n (%)	18 (53%)
PSQI, median (IQR)	10 (5–13)
PSQI > 5, n (%)	23 (68%)
ESS, mean ± SD	4.79 ± 4.73
ESS > 10, n (%)	5 (15%)
SCI, mean ± SD	16.44 ± 7.57
SCI > 16, n (%)	13 (38%)
SCI < 16, n (%)	21 (62%)

Abbreviations: ESS = Epworth Sleepiness Scale; MEQ = Morningness–Eveningness Questionnaire; PSQI = Pittsburgh Sleep Quality Index; SCI = Sleep Condition Indicator.

**Table 3:** Baseline characteristics of responders vs. non-responders.

Features	Non-Responders (n = 18)	Responders (n = 70)
Women, n (%)	16 (89%)	59 (84%)
Age, mean ± SD	51.1 ± 13.2	50.9 ± 8.8
MMDs, mean ± SD	26.1 ± 5.1	22.7 ± 5.7
DSMs, mean ± SD	18.9 ± 8.6	18.1 ± 7.5
Patients with medication overuse, n (%)	18 (100%)	67 (96%)
No. previous preventatives, mean ± SD	4.6 ± 1.6	3.9 ± 1.6
Patients with concomitant use of preventive therapies, n (%)	13 (72%)	37 (52%)
Patients treated with erenumab 70 mg, n (%)	13 (72%)	37 (53%)
Patients treated with erenumab 140 mg, n (%)	5 (28%)	33 (47%)

Abbreviations: DSMs = Days with Symptomatic Medications per month; MMDs = Monthly Migraine Days.

**Table 4:** Adverse events list.

Adverse Effects	No. Patients (%)
constipation	9 (10%)
swelling	4 (5%)
nausea	2 (2%)
cramps	3 (3%)
dizziness	2 (2%)
itch	2 (2%)
transient rash	1 (1%)
fatigue	2 (2%)
muscle aches	3 (3%)
insomnia	5 (6%)

## Data Availability

Data will be available after reasonable request to the corresponding author.
